# Rho GTPases as therapeutic targets in Alzheimer’s disease

**DOI:** 10.1186/s13195-017-0320-4

**Published:** 2017-12-15

**Authors:** Byron J. Aguilar, Yi Zhu, Qun Lu

**Affiliations:** 1 Department of Anatomy and Cell Biology, Brody School of Medicine at East Carolina University, Greenville, NC 27834 USA; 2 The Harriet and John Wooten Laboratory for Alzheimer’s and Neurodegenerative Diseases Research, Brody School of Medicine at East Carolina University, Greenville, NC 27834 USA

**Keywords:** Alzheimer’s disease, Rho GTPases, Cdc42, Rac1, RhoA, NSAIDs, AD mouse model, AD therapy

## Abstract

The progress we have made in understanding Alzheimer’s disease (AD) pathogenesis has led to the identification of several novel pathways and potential therapeutic targets. Rho GTPases have been implicated as critical components in AD pathogenesis, but their various functions and interactions make understanding their complex signaling challenging to study. Recent advancements in both the field of AD and Rho GTPase drug development provide novel tools for the elucidation of Rho GTPases as a viable target for AD. Herein, we summarize the fluctuating activity of Rho GTPases in various stages of AD pathogenesis and in several in vitro and in vivo AD models. We also review the current pharmacological tools such as NSAIDs, RhoA/ROCK, Rac1, and Cdc42 inhibitors used to target Rho GTPases and their use in AD-related studies. Finally, we summarize the behavioral modifications following Rho GTPase modulation in several AD mouse models. As key regulators of several AD-related signals, Rho GTPases have been studied as targets in AD. However, a consensus has yet to be reached regarding the stage at which targeting Rho GTPases would be the most beneficial. The studies discussed herein emphasize the critical role of Rho GTPases and the benefits of their modulation in AD.

## Background

Rho GTPases are an important target in human diseases such as Alzheimer’s disease (AD), cardiovascular, pulmonary, and neurological disorders, and cancers [1]. The various roles of Rho GTPases in AD pathogenesis have been extensively reviewed [2, 3]. It is widely known that AD pathology is characterized by the accumulation of β-amyloid (Aβ) plaques and neurofibrillary tangles (NFTs) [4], which leads to progressive loss and alterations in synaptic efficacy and damage at the synaptic terminal [5]. The dynamic regulation of actin polymerization plays a critical role in morphological changes in dendritic spines [6, 7]. The prominent regulators of actin polymerization are the Rho family of small GTPases that includes cell division cycle 42 (Cdc42), Ras-related C3 botulinum toxin substrate 1 (Rac1), and Ras homolog gene family, member A (RhoA) [8, 9]. Rho GTPase activity is regulated by guanine nucleotide exchange factors (GEFs) that stimulate the release of GDP, GTPase activating proteins (GAPs) that promote the hydrolysis of GTP, and guanine nucleotide dissociation inhibitors (GDIs) that sequester the protein. Rho GTPases play critical roles in dendritic spine morphogenesis and synaptic plasticity [10, 11]. Aberrant activity of the Rho GTPases, their regulators (GEFs, GAPS, and GDIs), and effectors in AD has been reviewed [2, 12]. However, the therapeutic potential of Rho GTPases in AD remains unclear. This is due to the limitations of previous methods/technology and the variety of experiments/models used to study Rho GTPases in AD. Herein, we summarize the fluctuating activity of Rho GTPases in various stages of AD pathogenesis and in several in vitro and in vivo AD models. The work discussed in this review serves as the foundation for Rho GTPases in AD, but a unified and more complete approach must be adopted to understand the intricacies of Rho GTPase signaling in AD.

## Rho GTPases in Alzheimer’s disease

Synaptic loss is a major contributor to cognitive impairment associated with AD [5, 13, 14], and changes in neuroplasticity over time may explain the late onset of AD [15]. It is not surprising that the Rho GTPase signaling pathway has been the subject of various studies given the synaptic changes often observed in AD. In addition to AD, Rho GTPases are also dysregulated in a variety of neurological diseases/disorders that show synaptic irregularity such as Huntington’s disease, Parkinson’s disease, amyotrophic lateral sclerosis, and schizophrenia [16, 17]. The various experiments with constitutively active and/or dominant negative mutants of Rho GTPases [18] and the opposing effects of Rho GTPase activity regulating primary neuron axonal branching/elongation have been reviewed [19]. The overall consensus is that Cdc42 and Rac1 activation stimulates axonal growth, spine formation, and dendritic branching, while RhoA activation is inhibitory [20, 21]. However, there are some conflicting reports of Rho GTPase activity in AD-related studies (reviewed in [16]). This highlights the complexity and variability of Rho GTPase activity in AD (Table 1). Additionally, this underscores the importance of developing novel models and approaches that examine Rho GTPase in AD signaling as a whole, rather than individually.

**Table 1:** Summary of Rho GTPase alterations in AD mouse models and AD patients

Model	Rho GTPase alterations	Reference
Tg2576 hAPP Swedish (KM670/671NL)	• 12- to 18-month-old, overall ↑ RhoA and ↓ Rac1 in brain • 18-month-old, RhoA ↓ synapse and ↑ distrophic neurites • 18-month-old, ↑ Rac1 in cortex	[26] [33] [37]
hAPP J20 hAPP Swedish, Indiana (V717F)	• 14-month-old, ↑ RhoA hippocampus	[38]
5 × FAD/Tg6799 hAPP Swedish, Florida (I716V), London (V717I); PSEN1 (M146L/L286V)	• 9- to 10-month-old, ↑ RhoA brain capillaries	[39]
AD patients	• ↓ Rac1 early stage AD • ↑ Cdc42 and Rac1 in select neuronal populations • ↓ Rac1 and PAK1 in frontal and occipital lobes • RhoA ↓ in neuropils, ↑ neurons, and colocalized with hyperphosphorylated tau leading to ↓ RhoA activity	[41] [3] [42] [33]

*AD* Alzheimer’s disease

### Rho GTPases and AD pathology

**Fig. 1:**
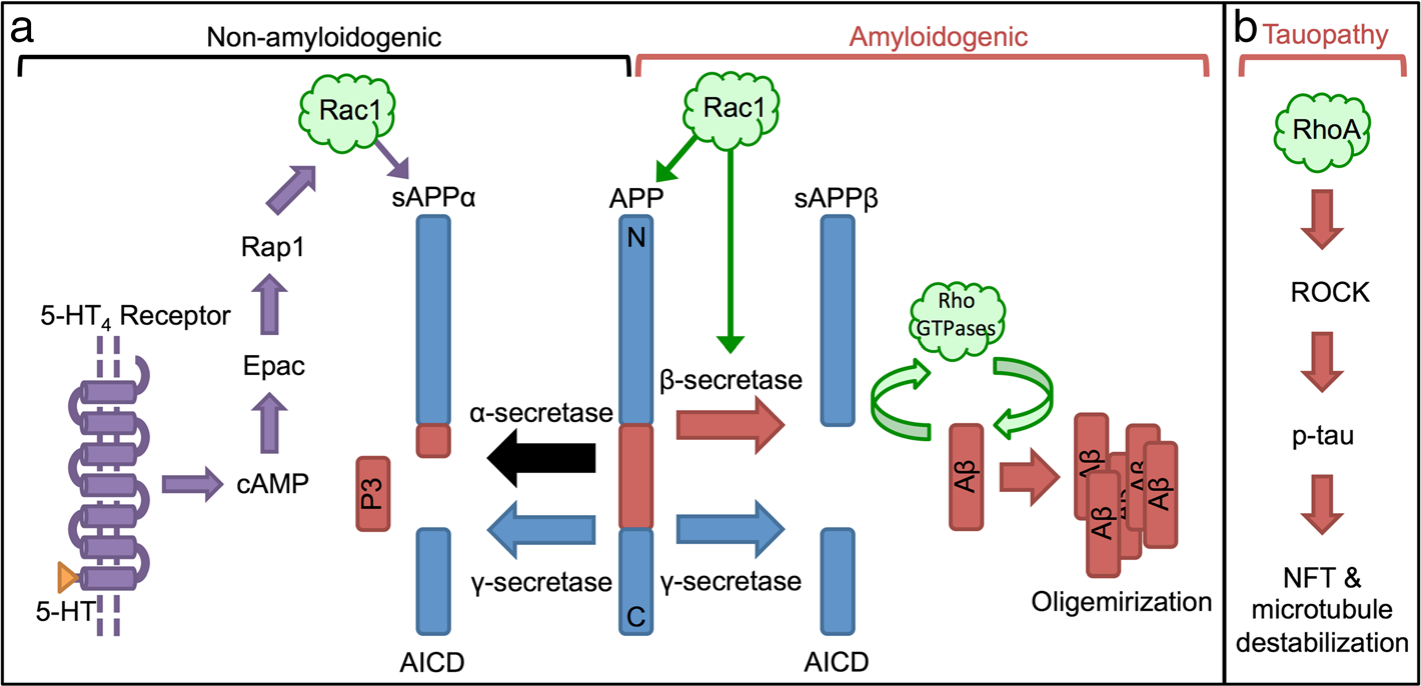
Rho GTPases and AD pathology. **a** Amyloid precursor protein (*APP*) can undergo amyloidogenic (*right*) or non-amyloidogenic (*left*) processing. In the amyloidogenic pathway, β-secretase cleavage results in the formation of soluble APPβ (*sAPPβ*). Cleavage by γ-secretase forms β-amyloid (*Aβ*) and amyloid precursor protein intracellular domain (*AICD*). Accumulation of Aβ leads to amyloid plaque formation. Several studies have reported the activation loop between Aβ and Rho GTPases (*green arrows*). Activated Rac1 can increase APP production and promote the amyloidogenic pathway by modifying β-secretase selectivity for APP (*green arrows*). In the non-amyloidogenic pathway, α-secretase cleaves within the Aβ region, which results in the formation of sAPPα. Cleavage by γ-secretase forms the P3 peptide and AICD. Activation of Rac1 via the 5-HT_4_/cAMP/Epac/Rap/Rac1 signaling cascade promotes the formation of sAPPα (*purple arrows*). **b** RhoA activates ROCK that can phosphorylate tau (Thr245 and Ser409) leading to neurofibrillary tangle (*NFT*) and microtubule destabilization. *5-HT* serotonin, *cAMP* cyclic adenosine monophosphate, *Epac* exchange proteins directly activated by cAMP, *Rap1* Ras-proximate-1/Ras-related protein-1, *RhoA* Ras homolog gene family, member A, *ROCK* Rho-associated protein kinase

Given that Rho GTPases are dysregulated in AD, several studies have examined the relationship between Rho GTPases, amyloid precursor protein (APP) synthesis, and β-amyloid (Aβ) production in various cell lines. For example, in mouse primary hippocampal neurons, Rac1 inhibition negatively regulates APP gene synthesis [22] as well as attenuates Aβ_42_ production by altering γ-secretase substrate selectivity and increasing the processing of Notch1 over APP [23]. In COS-7 cells (fibroblast-like cells derived from monkey kidney tissue), dominant negative Rac1 diminished γ-secretase activity resulting in decreased production of the APP intracellular domain and accumulation of the C-terminal fragments [23]. In PC12 cells (derived from rat adrenal gland), Aβ_42_ treatment activated RhoA and decreased neuronal survival by inhibiting protein tyrosine phosphatase 1B (PTP1B). Thus, Rho GTPases appear to contribute to the increase in Aβ and resulting neurotoxicity (Fig. 1).

While the role of Rac1 appears to be clearer, Cdc42 may be cell specific. Both Rac1 and Cdc42 activity are increased in hippocampal neurons treated with Aβ_42_ [24]. Meanwhile, SN4741 cells (a dopaminergic neuronal cell line of embryonic substantia nigra origin derived from the mouse) treated with Aβ_42_ stimulated Rac1 activation and had no effect on Cdc42 and RhoA activation [25]. In contrast, Aβ_40_ and Aβ_42_ activated RhoA, inhibited Rac1, and decreased neurite outgrowth of SH-SY5Y (human neuroblastoma) cells [26]. RhoA inhibition via a Rho-associated protein kinase (ROCK) inhibitor or expression of constitutively active Rac1 attenuated the effects of Aβ_40_. Interestingly, activation of RhoA has also been seen in a platelet model, which processes amyloid similar to neurons [27]. The Aβ_25–35_ fragment exhibits increased solubility and similar biological effects to Aβ_42_ [28, 29]. Platelets exposed to Aβ_25–35_ increased RhoA activation, increased phosphorylation of myosin light chain (MLC) and MLC phosphatase, and increased platelet aggregation and clot retraction [30]. Similarly, these effects were attenuated when treated with a ROCK inhibitor.

In addition to the canonical activators of Rho GTPases (i.e., epidermal growth factor receptor (EGFR) and Ras), other pathways such as the estrogen receptor have been shown to activate Rho GTPases (reviewed in [31]). These findings indicate that activation of Rho GTPases by other signaling cascades may potentially elicit variable effects. For example, activation of Rac1 by the serotonin 5-HT_4_ receptor via the 5-HT_4_/cAMP/Epac/Rap1/Rac1 signaling axis stimulates the non-amyloidogenic pathway [32]. The authors characterize activation of Rac1 as cyclic adenosine monophosphate (cAMP)-regulated and PKA-independent while RhoA and Cdc42 are cAMP-regulated and PKA-dependent. Activation of Rac1 was observed following stimulation with serotonin in Chinese hamster ovary (CHO) cells, mouse primary neurons, and in the human IMR32 neuroblastoma cell line [32]. Given that activation of RhoA and Cdc42 was not reported, the activity of these Rho GTPases is unclear.

Although there are not as many reported studies, Rho GTPases are also implicated in tau pathology. In human AD cortex and hippocampus, immunohistological analysis revealed that only RhoA colocalized with hyperphosphorylated tau [33]. Alternatively, in an AD mouse model, RhoA did not colocalize with hyperphosphorylated tau, which the authors attributed to the lack of tau pathology in the model [33]. Thus, the absence of tau in the animal model indicates that a more complete model of AD is required to evaluate the roles of Rho GTPases in tauopathy. Indeed, RhoA/ROCK pathway phosphorylates tau at several sites such as Thr245 and Ser409 in COS7 cells transfected with tau [34]. Site-directed mutagenesis studies revealed a decrease in microtubule assembly, thus confirming the functional significance of these phosphorylation sites (Thr245 and Ser409).

### Rho GTPases and AD mouse models

Tg2576 is the most commonly used AD transgenic mouse model. Tg2576 is a transgenic mouse model expressing the Swedish mutation (KM670/671NL) of the human amyloid precursor protein (hAPP) that causes memory deficits and plaque accumulation with age [35, 36]. The following studies focus on Rho GTPases using the Tg2576 mouse model, which seem to indicate fluctuating activity in certain regions of the brain. For example, in 12- to 18-month-old Tg2576 mice, there was an overall increase in RhoA and decrease in Rac1 activity in the brain compared to age-matched controls [26]. In 18-month-old Tg2576, RhoA expression decreased in synapses and increased in dystrophic neurites, suggesting altered subcellular targeting of RhoA [33]. Another study showed an increase in Rac1 immunoreactivity localized near Aβ plaques in cortex sections derived from 18-month-old Tg2576 [37].

Rho GTPase activity in other AD mouse models has also been examined. In 14-month-old hAPP J20 mice with Swedish and Indiana (V717F) mutations, RhoA activity was significantly increased in the hippocampus [38]. This increase of RhoA was also observed in 9- to 10-month-old 5 × FAD/Tg6799 mice brain capillaries [39]. 5 × FAD harbor hAPP Swedish, Florida (I716V), and London (V717I) mutations, as well as PSEN1 (M146L and L286V) mutations [40].

### RhoA vs Cdc42/Rac1 in AD patients

There have also been studies that focused on Rho GTPase activity in AD patients. In select neuronal populations in AD brains, Cdc42 and Rac1 expression was elevated when compared to age-matched controls [3]. Additionally, Cdc42 and Rac1 overexpression in both neurons with NFTs and normal neurons with cytoskeletal abnormalities led the authors to speculate that increased expression may be a prerequisite to AD pathogenesis [3]. On the other hand, Rac1 expression is decreased in early-stage AD patients [41]. Rac1 and PAK1 decreased in the frontal and occipital lobes in AD patients when compared to age-matched controls [42]. There was also a negative correlation between Rac1 and phosphorylated tau but no correlation with Aβ was observed. The authors also reported a Rac1 and PSD-95 correlation in the frontal but not occipital lobe of AD patients [42]. Immunohistological studies of RhoA in AD brains showed altered RhoA subcellular localization, whereas Cdc42 and Rac1 remained unchanged. RhoA staining decreased in the neuropil, increased in neurons, and colocalized with hyperphosphorylated tau leading to decreased RhoA activity [33]. Taken together, the activity levels of Rho GTPases in AD patients appear to play a pivotal role in pathogenesis and may vary according to disease stage and/or be localized to specific neuronal populations.

## Development of Rho GTPase pathway modulators

Targeting the various Rho GTPases and their signaling pathways has reached various stages in drug development. Early studies revealed that activation of Rho GTPases enhanced learning and memory in mice. Meanwhile, inhibitors of Rho GTPases were also shown to attenuate AD pathology. Investigations of RhoA focused on nonsteroidal anti-inflammatory drugs (NSAIDs) early on and then ROCK for more specific inhibition. Rac1 studies used synthetic Rac1 inhibitors. To date, there has been no report of specifically modulating Cdc42 with small molecules in AD studies. Altogether, these pharmacological tools have aided in probing the specific functions and highlighted the potential of the Rho GTPases as therapeutic targets in AD. Table 2 summarizes the AD-related effects of pharmacological inhibition of Rho GTPases.

**Table 2:** Summary of AD-related effects of Rho GTPase pharmacological inhibition

Treatment	Models	Effects	Reference
Rac1 NSC23766, 6-MP, EHT1864	• Hippocampal neurons from 15-day-old embryonic ICR mice • HEK293, RACT17N (negative mutant), and siRNA	Decreased APP protein and mRNA levels.	[22]
	• SN4741 cells • Primary neuron cultures from the cortical lobes of E18 Sprague–Dawley rat embryos, hippocampal and entorhinal	Blocked Aβ_42_-associcated neuronal death.	[25]
	• SH-SY5Y, HEK293-swAPP, and HEK293-BACE cells	Decreased Aβ protein level. Did not affect BACE, α-secretase, and Notch1. Inhibited γ-secretase, indirectly.	[52]
NSAIDs Ibuprofen, cicoflenac, indomethacin, fenoprofen, sulindac sulfide, meclofen, flurbiprofen	• SH-SY5Y-swAPP • PDAPP mice	Decreased Aβ_42_ production.	[53]
	• CHO WT-APP and PS1-M146L. • HS683-swAPP and PSN • 3-month-old Tg2576	Decreased Aβ_42_ formation and increased Aβ_38_. Acute dosing in mice decreased Aβ_42_ brain levels	[54]
	• 10-month-old Tg2576	Reduced CNS inflammatory response and amyloid plaque pathology.	[55]
	• Neuro2a N2a NL/N-swAPP and NotchΔE	Decreased Aβ_42_, no effect on Aβ_40_ and Notch1.	[62]
	• H4-swAPP • CHO-sw/loAPP • 3-month-old female Tg2576 mice	Decreased Aβ42 in a γ-secretase-dependent manner both in vitro and in vivo.	[63]
Indomethacin	• Mild/moderate AD patients	Slight improvement in cognitive tests: Mini-Mental State Examination, Alzheimer's Disease Assessment Scale, Boston Naming Test, and Token Test.	[56]
R-Flurbiprofen	• Mild AD patients	Ineffective at preventing/delaying loss of cognition or function.	[64]
RhoA/ROCK Y27632, SR3677, HA-1077	• CHO with both wild-type (WT) human APP751 and human mutant PS1.	Inhibited Aβ_42_ production.	[67]
	• Platelets	Inhibited cytoskeletal reorganization following activation with Aβ_25–35_.	[30]
	• SH-SY5Y cells • ~2-month-old PDAPP	Lowered Aβ_42_ levels in vitro and in vivo.	[53]
	• SH-SY5Y, HEK293, and primary cortical neurons from embryonic day 17 mouse embryos • 3-month-old 5 × FAD	Inhibited BACE activity and Aβ production.	[68]

*Aβ* β-amyloid, *AD* Alzheimer’s disease, *APP* amyloid precursor protein, *BACE* beta-site APP cleaving enzyme, *CNS* central nervous system, *CHO* Chinese hamster ovary, *NSAIDs* nonsteroidal anti-inflammatory drugs

### Cytotoxic necrotizing factor 1

Cytotoxic necrotizing factor 1 (CNF1) is a toxin isolated from *Escherichia coli* and originally reported over 30 years ago [43, 44]. It induces actin reorganization via activation of Rho GTPases [45]. The structure, specificity, and activity of CNF1 have been reviewed [46]. CNF1 catalyzes the deamidation of a glutamine residue to impede GTP hydrolysis leading to persistent activation [47, 48]. Although CNF1 increases RhoA, Rac1, and Cdc42 GTPase activity, in vivo studies with mice showed that activation of Rac1 lasted longer than RhoA (28 days and ~10 days, respectively) [49]. Prolonged activation of Rac1 was also observed in rats [50]. Behavioral studies conducted with CNF1 are discussed later in this review.

### Rac1 inhibitors

The availability of Rac1 inhibitors has increased in the last decade and has resulted in several studies that focused on elucidating the role and evaluating the therapeutic potential of Rac1 in AD. NSC23766 is the most commonly used Rac1 inhibitor, and prevents the Rac1-GEF interaction necessary for nucleotide exchange [51]. Inhibition of Rac1 with NSC23766 decreased APP and Aβ levels via APP gene regulation [22]. NCS23766 and 6-MP, another Rac1 inhibitor, also efficiently prevented Aβ_42_ peptide-induced cell death in SN4741 cells and in both primary neurons from the hippocampus and the entorhinal cortex [25]. Both NSC23766 and EHT1864, another Rac1 inhibitor, altered APP metabolism processing by selectively inhibiting γ-secretase metabolism [52].

### NSAIDs

Unlike Rac1, the unavailability of small molecule inhibitors makes direct modulation of RhoA in AD models more challenging. However, a few studies have evaluated the use of NSAIDs, which primarily function as cyclooxygenase (COX) inhibitors, to show that they may affect RhoA activity. NSAIDs such as sulindac sulfide, ibuprofen, and indomethacin were effective in lowering Aβ_42_ formation and inhibiting RhoA activity in SH-SY5Y cells transfected with the Swedish mutant APP695 and in the AD transgenic PDAPP mouse model [53]. These NSAIDs also decreased Aβ_42_ and increased Aβ_38_ formation in both CHO and human neuroglioma HS683 cells transfected with APP and presenilin mutants as well as in cultured Tg2576 mouse neurons [54]. In 10-month-old Tg2576 mice, chronic treatment with ibuprofen (for 6 months) reduced both the central nervous system (CNS) inflammatory response and amyloid plaque pathology [55]. A clinical trial with indomethacin showed a slight improvement in various cognitive tests in patients with mild/moderate AD [56]. Interestingly, it appeared as if only COX-positive NSAIDs were able to inhibit Aβ formation. However, studies with COX-knockout and several NSAID COX inhibitors indicated that the anti-AD effects of NSAIDs were independent of the COX signaling pathway [57].

Several reports have proposed AD-related mechanisms of action for NSAIDs. Since NSAID activity against Aβ_42_ correlated with RhoA inhibition [58], and Y27632 decreased Aβ_42_ and increased Aβ_38_ similar to NSAID treatment [53], the RhoA-ROCK pathway was identified as a potential mechanism for NSAID activity. Alternatively, NSAIDs may be acting on γ-secretase to alter APP processing. NSAIDs appeared to alter only AD-related APP processing and not the metabolism of Notch, another known substrate of γ-secretase [59, 60]. Sulindac sulfide directly inhibits γ-secretase activity with higher efficacy at inhibiting Aβ_42_ formation than Aβ_40_ and Notch processing [61]. R-flurbiprofen is a COX-negative NSAID that can be converted to the COX-positive S-enantiomer in the body [62]. Both enantiomers of flurbiprofen were effective at inhibiting Aβ_42_ formation by targeting γ-secretase [63]. However, a randomized controlled trial with R-flurbiprofen was ineffective in patients with mild AD [64].

### ROCK inhibitors

Given the lack of effective small molecules that directly inhibit RhoA, ROCK inhibitors have been used to study the effects of RhoA-ROCK inhibition. The use of ROCK inhibitors in diseases such as hypertension, inflammation, and atherosclerosis has been reviewed elsewhere [65]. Y27632 was originally reported by Uehata and colleagues [66] as an antihypertensive and is perhaps the most commonly used small molecule inhibitor to study the RhoA-ROCK pathway in AD. 293-APP695sw cells transfected with activated or inactivated mutant ROCK1 did not alter Aβ levels [67]. However, ROCK inhibitors Y27632 or HA-1077 were effective at lowering Aβ levels, but were not selective for Aβ_42_ [67]. Treatment of 2-month-old PDAPP mice with Y27632 lowered brain Aβ_42_ levels [53]. Interestingly, Aβ levels were increased following ROCK1 knockdown in SH-SY5Y cells, but were decreased after ROCK2 knockdown [68]. SR3677, a selective ROCK2 inhibitor, and not Y27632 suppressed β-site APP cleaving enzyme 1 (BACE1) enzymatic action and diminished production of Aβ in 3-month-old 5 × FAD AD mouse brain [68]. While the use of ROCK inhibitors in AD-related clinical trials is limited, the identification and development of over 170 ROCK inhibitors and their therapeutic potential in neurodegeneration and neurotrauma have been reviewed elsewhere [69, 70].

### Cdc42 and/or Rac1 inhibitors

Recent drug design and development efforts have led to the identification of several novel Cdc42 inhibitors, which we have recently reviewed [31]. A high-throughput biological screening led to the discovery of CID2950007/ML141, a nucleotide binding inhibitor that is selective for Cdc42 [71]. AZA1 is a dual Cdc42 and Rac1 inhibitor originally designed to inhibit Rac1-GEF interactions based on the Rac1 inhibitor NSC23766 [72]. We have previously reported the identification of several Cdc42 inhibitors (ZCL compounds) using high-throughput *in silico* analysis and biological screening [73]. ZCL compounds modify the binding of Cdc42 and intersectin, a Cdc42-specific GEF. Interestingly, several NSAIDs have been identified to inhibit Cdc42 activity. Nonselective COX NSAIDs such as naproxen and ketorolac have been reported to inhibit Cdc42 and Rac1 [74]. Thus, the advent of specific pharmacological tools targeting Cdc42 provides a viable means to study Cdc42 and may enhance our understanding of its role in AD pathogenesis.

## Rho GTPases modify behavior in AD mouse models

The morphology of dendritic spines, which is closely regulated by Rho GTPases (RhoA, Rac1, and Cdc42), plays an important role in neuronal development and function [75]. Mutations in Rho-linked genes cause human intellectual disability (ID) [76] and further implicate the involvement of Rho GTPases in neural diseases. Clinical observations also indicate that patients who have neural diseases often display a variety of behavior disorders [77]. Therefore, several behavior studies have been carried out to investigate the link between Rho GTPase activity and behavior alteration (Table 3). In this section, we highlight the changes in behavior that result from the regulation of Rho GTPases in various mouse models. The following transgenic mouse models underscore the importance of Rho GTPases in modifying behavior and identify key characteristics that are changed. Currently, only the ROCK inhibitor Y27632 has been used to study behavior in an AD mouse model. With the recent advancements in pharmacological tools that target Rho GTPases, we anticipate a growth in behavioral studies following pharmacological Rho GTPase modulation.

**Table 3:** Rho GTPase modulation and behavioral modifications

Mouse model	Treatment	Behavioral modification	Reference
C57BL/6, male, 2-month-old	CNF1	(1) Enhanced associative emotional memories in the fear conditioning test. (2) Improved spatial learning in the water maze paradigm.	[49]
ICR, male, 2-month-old	Y27632	(1) Increased anxiety-related behaviors: a. Spent less time on the open arms of elevated plus-maze test. b. Frequently froze in the fear-conditioning test. c. Decreased head-dipping behavior in the hole-board test. (2) No spatial learning alteration in the Y-maze test.	[89]
TgCRND8, male/female, 4-month-old	CNF1	(1) Decreased locomotor hyperactivity in the open field test. (2) Corrected the deficit of spatial learning in the water maze paradigm.	[78]
ApoE4, male/female, 12-month-old	CNF1	(1) Improved spatial learning memory in the water maze paradigm. (2) Enhanced the non-spatial learning memory in passive avoidance test. (3) CNF1 treatment did not induce anxiety behaviors. (No significant difference was observed in the elevated plus maze and open field test)	[81]
MeCP2-308, female, 12-month-old	CNF1	(1) Reversed the spatial reference memory deficit in the Barne maze test.	[83]
*αPIX*/*ARHGEF6* knockout, male, ~6-month-old	None	(1) Slow adaptation of new target in the water maze test. (2) Intact spatial learning memory was observed in the eight-arm radial maze test. (3) Overly increased activity to the unknown object in the open field test. (4) Displayed a deficit in the complex spatial learning ability in the IntelliCage test.	[88]
*Arg* ^*–/–*^ *,* male, ~4-month-old	None	(1) Failed to distinguish the novel object in the object recognition task.	[86]

*CNF1* cytotoxic necrotizing factor 1

### Rho GTPase activation with CNF1

Several studies have reported the use of CNF1 to improve learning behavior in both wild-type (WT) and transgenic mouse models. In 2-month-old male C57BL/6 mice, intracerebroventricular (icv) injection of CNF1 enhanced fear conditioning, which indicated an improvement in associative learning [49]. Furthermore, CNF1-treated mice also showed improved spatial learning in the water maze task. In 4-month-old TgCRND8 mice that carry the mutated hAPP gene (KM670/671NL + V717F), icv injection of CNF1 attenuated impairments of spatial learning in the water maze paradigm and decreased locomotor hyperactivity in the open field test [78]. Untreated TgCRND8 mice displayed increased locomotor hyperactivity and deterioration of cognitive functions indicated by mild deficits in place learning and impairment in reversal learning (after displacement of the escape platform) at the water maze task.

The apolipoprotein E type 4 (apoE4) AD model exhibits the late-onset of Aβ deposits with enhanced oligomerization and/or decreased clearance [79, 80]. In apoE4 and not in apoE3 (neuroprotective) mice, CNF1 reduced Rho GTPase activation in the hippocampus but increased in the frontal cortex [81]. CNF1-treated 12-month-old apoE4 mice showed improved spatial learning in the water maze test and enhanced non-spatial learning in the passive avoidance test. Furthermore, CNF1 treatment did not induce anxiety behaviors as no significant difference was observed between control and treatment groups of mice in either the elevated plus maze or open field test.

Rho GTPase activation via CNF1 has also been shown to benefit other intellectual disability studies such as Rett syndrome (RTT), which is a severe X-linked neurodevelopmental disease affecting females [82]. Researchers found that 12-month-old MeCP2-308 female mice, a RTT mouse model, demonstrated a deficit in spatial memory in the Barne maze test [83]. Treatment with CNF1 restored WT-like spatial memory in MeCP2-308 female mice.

### GTPase inhibition

Tyrosine-protein kinase ABL2, also known as Arg, is a member of the Abl family nonreceptor tyrosine kinases. Unlike CNF1, Arg inhibits RhoA via its substrate p190RhoGAP [84]. Arg, p190RhoGAP, and RhoA are expressed in dendritic spines [85], which suggest that Arg/p190RhoGAP signaling may play a role in the stability of dendritic spines. After conducting the object recognition task on Arg^–/–^ mice, researchers noticed that 3- to 4-month-old male Arg^–/–^ mice exhibited no preference for the novel object, which indicated a deficit in hippocampus-dependent behavior [86]. Interestingly, both postnatal day 21 WT and Arg^–/–^ mice showed no difference in the object recognition task. However, 2-month-old Arg^–/–^ mice displayed a decreased level in exploration of the novel object when compared to the WT mice. The in vitro analysis of hippocampal CA1 pyramidal neurons from Arg^–/–^ mice indicated that neural dendrites developed normally at postnatal day 21. Dendritic spines did not reach morphological maturation by postnatal day 42, which further supported the age-dependent behavior change in postnatal day 21 and 2-month-old Arg^–/–^ mice [86].

Mutation of the *ARHGEF6* gene, which encodes αPIX/Cool-2, a GEF for Rac1 and Cdc42, has been found to cause X-linked ID in humans [87]. In 4- to 6-month-old αPIX/ARHGEF6 knockout mice, inhibition of Rac1 and Cdc42 activity induced slow adaptation to a new target in the water maze task and impaired complex spatial learning in the IntelliCage study [88]. However, intact spatial learning behavior was preserved in 6-month-old αPIX/ARHGEF6 knockout mice in the eight-arm radial maze test.

Pharmacological inhibition of RhoA/ROCK has also been studied. After icv injection of Y27632 to 2-month-old male ICR mice, no spatial learning deficit was observed in the Y-maze test, but there was an increase in anxiety-related behaviors. Y27632 treatment caused mice to spend less time in the open arms of the elevated plus-maze test, freeze more frequently than saline-treated mice in the fear conditioning test, and significantly suppressed head-dipping behavior in the hole-board test [89].

## Challenges and future perspectives

Mounting evidence suggests that AD is a multifaceted disease and that Rho GTPase modulation, at least in part, can attenuate the effects of AD-related pathology. Several studies have elucidated the therapeutic potential of Rho GTPases, but the exact mechanisms of some pharmacological tools remain unclear. While the beneficial effects of Rho GTPase modulators correlated with their respective target, the use of NSAIDs and their ultimate target are still uncertain. NSAIDs appear to elicit an anti-AD effect that is related to Rho GTPases and independent of COX. Alternatively, statins have also been reported to produce anti-AD effects by inhibiting Rho GTPases [90, 91]. The effects of several classes of pharmacological agents to inhibit Rho GTPase and their anti-AD effects further underscores the therapeutic potential of Rho GTPases in AD. Further testing to characterize the exact mechanism of action of these pharmacological tools will aid in their use for future studies.

The work summarized in this review points to a strong connection between Rho GTPases and animal behavior. Improved learning and memory following CNF1 treatment indicates that activation of Rho GTPases has a beneficial effect. Since activation of Rac1 and Cdc42 are associated with neurite growth and RhoA is associated with pruning, the exact changes due to CNF1 treatment need to be studied. Due to the differences in turnover rates of Rho GTPases, Rac1/Cdc42 will remain modified/activated after CNF1 treatment much longer than RhoA (28 and ~10 days, respectively) [49]. In contrast, we have also discussed the benefits of Rho GTPase inhibition (i.e., decreased Aβ levels, decreased neurotoxicity, and improved behavior) with pharmacological tools. Altogether, these studies paint the picture of a delicate balance of Rho GTPase activity and the benefits of restoring this balance.

Given the complexity of Rho GTPase signaling, it is critical to dissect each cascade at various and specific stages of AD pathogenesis to effectively evaluate their therapeutic potential. Recent advancements in the development of Rho GTPase modulators have led to later generations of RhoA/ROCK and Rac1 inhibitors as well as the very first Cdc42-specific modulators (ZCL compounds). In addition to advancements in pharmacological tools, AD research has also seen an increase in disease animal models. For example, the triple transgenic (3 × Tg)-AD mouse model that harbors mutations of APP, presenilin-1, and microtubule-associated protein tau [92] emphasizes the accelerated AD-like pathogenesis and the overall disease phenotype. The use of such models may effectively address limitations associated with earlier models that lack complete AD pathology. Our preliminary data with the ZCL compounds and the 3 × Tg-AD mouse model that focused on studying activities of daily living (ADL) revealed beneficial modifications. ADL has become a crucial tool for preclinical behavioral screening of neurodegenerative diseases [93], especially in AD. Studies on the effects of ZCL compounds on AD pathology are currently ongoing.

Due to the crosstalk and dysregulation of Rho GTPases, it would also be necessary to determine the role of Rho GTPase regulators (GEFs, GAPS, and GDIs) in AD. Indeed, Rho GTPase regulators and effectors have been implicated in AD (reviewed in [2, 12]). We have recently reviewed the activation of Cdc42 in Ras-related cancers as well as the effectors and regulators [31] given that oncogenic pathways are also activated in AD [3]. More recent studies have identified and characterized certain Rho GTPase regulators as key components in AD. For example, intersectin (ITSN) is a specific GEF for Cdc42 and is implicated in AD, Huntington’s disease, and Down’s syndrome [8, 94, 95]. Overexpression of ITSN contributes to AD by altering endocytosis [96] and increasing mitogen-activated protein kinase (MAPK) signaling [97–100]. This line of evidence specifically supports targeting the ITSN-Cdc42 signaling pathway as a potential target in AD. We have reviewed the development and use of current pharmacological tools that target Cdc42 and its regulators and effectors [31]. In particular, the ZCL compounds, which were designed to inhibit ITSN-Cdc42 interactions [73], may serve as a valuable tool to determine the effects of ITSN-Cdc42 inhibition in AD.

## Conclusions

Overall, the studies discussed in this review highlight the critical functions of Rho GTPases in AD. Rho GTPases clearly play an essential role that appears to fluctuate during disease progression and/or location. These reports underscore the importance of Rho GTPases and reveal that our understanding of their roles in AD is still not complete. The use of more complete AD models (i.e., 3 × Tg-AD mouse model), specific assays that test for each Rho GTPase activation over time (i.e., 4–18 months), and specifying the region of the brain (i.e., hippocampus and cortex) will help elucidate the role of Rho GTPases in AD. Further characterization of Rho GTPases and evaluation of Rho GTPase modulators will both define and fill an area of AD-related therapy that may ultimately lead to the development of novel pharmacological tools for future clinical trials.

## Abbreviations

3 × Tg-AD: Triple-transgenic Alzheimer’s disease
5-HT: Serotonin
Aβ: β-Amyloid
AD: Alzheimer’s disease
ADL: Activities of daily living
apoE4: Apolipoprotein E type 4
APP: Amyloid precursor protein
BACE1: β-Site APP cleaving enzyme 1
cAMP: Cyclic adenosine monophosphate
Cdc42: Cell division cycle 42
CHO: Chinese hamster ovary
CNF1: Cytotoxic necrotizing factor 1
COX: Cyclooxygenase
Epac: Exchange proteins directly activated by cAMP
GAP: GTPase activating protein
GDI: Guanine nucleotide dissociation inhibitor
GEF: Guanine nucleotide exchange factor
hAPP: Human amyloid precursor protein
icv: Intracerebroventricular
ID: Intellectual disability
ITSN: Intersectin
MAPK: Mitogen-activated protein kinase
MLC: Myosin light chain
NFT: Neurofibrillary tangle
NSAID: Nonsteroidal anti-inflammatory drug
PTP1B: Protein tyrosine phosphatase 1B
Rac1: Ras-related C3 botulinum toxin substrate 1
Rap1: Ras-proximate-1 or Ras-related protein 1
RhoA: Ras homolog gene family, member A
ROCK: Rho-associated protein kinase
RTT: Rett syndrome
WT: Wild-type
